# Potential novel tick-borne Colpodella species parasite infection in patient with neurological symptoms

**DOI:** 10.1371/journal.pntd.0006546

**Published:** 2018-08-02

**Authors:** Jia-Fu Jiang, Rui-Ruo Jiang, Qiao-Cheng Chang, Yuan-Chun Zheng, Bao-Gui Jiang, Yi Sun, Na Jia, Ran Wei, Hong-Bo Liu, Qiu-Bo Huo, Hong Wang, Michael E. von Fricken, Wu-Chun Cao

**Affiliations:** 1 State Key Laboratory of Pathogen and Biosecurity, Beijing Institute of Microbiology and Epidemiology, Beijing, P. R. China; 2 Mudanjiang Forestry Central Hospital, Mudanjiang, P. R. China; 3 Division of Infectious Disease, Duke University Medical Center, Durham, North Carolina, United States of America; 4 Department of Global and Community Health, George Mason University, Fairfax, Virginia, United States of America; University of California San Diego School of Medicine, UNITED STATES

## Background

*Colpodella* is a close relative of the phylum Apicomplexa, which includes *Babesia*, *Plasmodium*, and others [1]. Recently, the *Colpodella* spp. strain human erythrocyte parasite (HEP) was identified in a patient in the Yunnan Province, Southwestern China [2], which shared some common features with cases of *Babesia*, like anemia, elevated reticulocyte counts, and lactate dehydrogenase levels. Here, we report another novel *Colpodella* spp. infection, which manifested differently than those patients and may be responsible for reported neurological symptoms, warranting further investigations.

## Case report

On May 23, 2013, a 55-year-old female was admitted to Mudanjiang Forestry Central Hospital, a sentinel hospital for tick-borne diseases located in Heilongjiang Province, Northeast China, with a chief complaint of dizziness, gait disturbance, and headache. Two weeks before admission, an engorged adult tick was removed from her supraclavicular fossae (S1 Fig). Eleven days after tick removal, she sought help at a local clinic due to fever (39.0 °C) and headache, where she received supportive treatment with compound paracetamol tablets for two days with no clinical improvement and persistent high fever up to 42.0 °C.

Upon admission, a routine exam showed a body temperature of 39.5 °C, a blood pressure of 125/70 mm Hg, a pulse rate of 60 beats/min, and a respiration of 18 breaths/min. The neurological check revealed moderate nuchal rigidity. No ulceration or exudation was observed around the tick bite location, nor were any erythematous lesions found on her trunk. A routine blood test showed that white blood cell (WBC) (7.6 × 10^9^/L) and red blood cell (RBC) (4.6 × 10^12^/L) levels were both in normal range, while the neutrophil—granulocyte proportion was substantially elevated (91.6%), along with 64.136 mg/L of C-reactive proteins (CRPs), indicating an inflammatory response. Laboratory tests of blood showed 53.1 U/L for alanine aminotransferase, 54.3 U/L for aspartate transaminase, and 78.0 U/L for gamma-glutamyl transferase in the blood that was collected on the day of admission, with 0.15 g/L protein detected in urine. Cerebrospinal fluid (CSF) tests revealed 0.4 g/L of protein, 4.28 mmol/L of glucose, and 125.3 mmol/L of chloride chloridate, with no WBC or RBC in the CSF, which was collected at four days after hospitalization and presented visually crystal clear and faint yellow. The routine test for the antibody of *Borellia* spp. and tick-borne encephalitis virus (TBEV) in CSF was routinely conducted in hospital once they collected the CSF sample for patients with recent tick bite history. The patient tested negative for TBEV but positive for immunoglobulin G (IgG) antibody against *Borrelia burgdorferi* sensu lato (sl) at titer of 1:256 in the serum, leading to a presumptive diagnosis of Lyme disease.

During her 10-day admission, the patient was administered doxycycline (100 mg/twice daily) [3]. Samples, including blood and CSF, were stored in liquid nitrogen and were transported to the Beijing Institute of Microbiology and Epidemiology at the end of July 28, 2013, for further testing. Specific PCR assays targeting *B*. *burgdorferi* sl, *Babesia* spp., *Anaplasma* spp., *Ehrlichia* spp., and *Rickettsia* spp were conducted for both blood and CSF samples. Except for the PCR assay targeting *Babesia* spp. in the CSF sample, all were negative. Furthermore, the recovered 1,627-bp sequence did not closely match any known characterized *Babesia* species [4]. Phylogenetic analysis revealed the nucleotide sequence was relatively close to *Colpodella* spp. with 89.0%–90.0% similarity (S2 Fig). The sequence was submitted to GenBank under accession No. KT364261, and we provisionally nominated it *Colpodella* spp. Heilongjiang (HLJ) strain. On April 15, 2015, almost two years after initial infection, no *Colpodella* spp. HLJ strain was detected, indicating no chronic or relapsing infection.

In addition, we conducted a screening for *Colpodella* spp. in 474 host-seeking adult *Ixodes persulcatus* collected in woodlands around the patient’s living area in the Mudanjiang area in 2015 with the same PCR assay, followed by direct sequencing, and subsequently found two *I*. *persulcatus* ticks positive for *Colpodella* spp. The positive amplicon was then cloned and sequenced. After sequence analysis, we identified two distinct sequences (GenBank accession No. KT600661, KT60062) that were 93.8% identical to each other, providing the first evidence of *Colpodella* spp. in *I*. *persulcatus* ticks found in China. Compared to the *Colpodella* spp. HLJ strain from our suspected clinical case (accession No. KT364261), the tick strains only shared an 88.0%–89.0% identity (S2 Fig). The detection of *Colpodella* spp. DNA in both the CSF of a patient with tick bite history along with isolation in ticks from the same region warrants deeper investigations.

## Discussion

The study was approved by the ethics committee of Mudanjiang Forest Central Hospital in accordance with the medical research regulations of China. The patient provided written informed consent. Although the patient’s clinical manifestation showed high probability of an infection with TBEV, the absence of specific IgG antibodies against TBEV and lack of elevated cerebrospinal cell counts in either the acute or convalescent stage serve to exclude TBEV infection. Despite observing elevated titers for *B*. *burgdorferi*, the absence of elevated protein and cells in the CSF, accompanied by negative PCR results for *B*. *burgdorferi* sl in both peripheral blood and CSF, suggests that this was not likely an acute infection of Lyme neuroborreliosis [5]. Based on the available data, Lyme neuroborreliosis and tick-borne encephalitis are unlikely to be the cause for the clinical presentation, while the presence of the *Colpodella* spp. HLJ strain, detected in the patient’s CSF, may have been responsible for the patient’s neurological symptoms. Furthermore, no erythrocytes or leukocytes were observed in the cerebrospinal fluid, illustrating that the *Colpodella* spp. HLJ strain may be free living, rather than parasitic in erythrocytes or leukocytes; however, no *Colpodella* organisms were visualized in the CSF. Laboratory contamination in this circumstance is highly unlikely, as *Colpodella* spp. are uncommon in human samples and the natural environment, with no *Colpodella* spp.-associated experiments conducted in our lab prior to this study. Additionally, preparation and amplification occurred in separate rooms to minimize the possibility of contamination.

After screening *Colpodella* spp. in host-seeking adult *I*. *persulcatus*, we detected *Colpodella* spp.-related sequences in *I*. *persulcatus*, which may possibly serve as the tick vector for this organism. Despite the unavailability of the biting tick in this study, based on the available data, we may deduce that the parasite potentially is transmitted through tick bite, which will require additional research to confirm. Thus, more efforts must be devoted to investigating the transmission and pathogenicity of *Colpodella* spp. in China and abroad.

**Key Learning Points**

- Physicians should consider *Colpodella* spp. infection in patients presenting with clinical features similar to cases of *Babesia*.
- Additional screening should occur in patients with a recent history of tick bites.
- The diagnosis of *Colpodella* spp. in patients currently depends on molecular methods.
- More efforts must be devoted to investigating the transmission and pathogenicity of *Colpodella* spp. in China and abroad.

## Supporting information

S1 Fig Timeline of the clinical course of the patient in 2013. (TIF)

S2 Fig Phylogenetic analysis based on the 18s rRNA gene of *Colpodella* spp. (TIF)
