# Supplementary figures and images for: Potential novel tick-borne Colpodella species parasite infection in patient with neurological symptoms
Source: PLoS Negl Trop Dis. 2018 Aug 2;12(8):e0006546. doi: 10.1371/journal.pntd.0006546 (PMC6071948; doi:10.1371/journal.pntd.0006546)

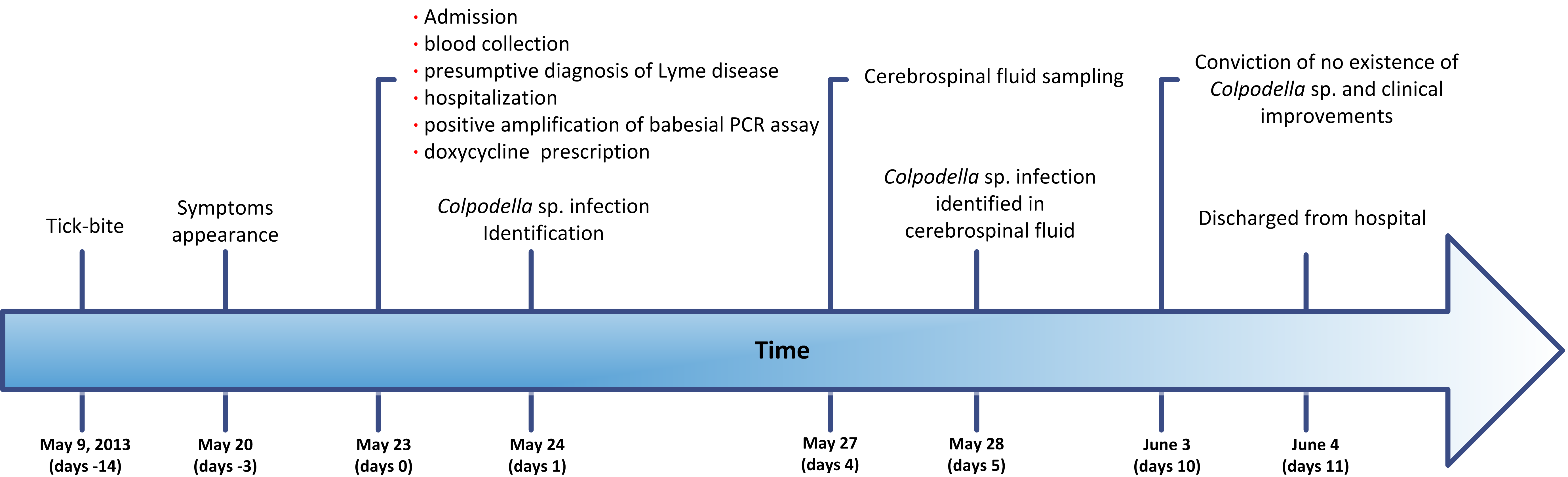

Supplement: S1 Fig — (TIF) [file pntd.0006546.s001.tif]

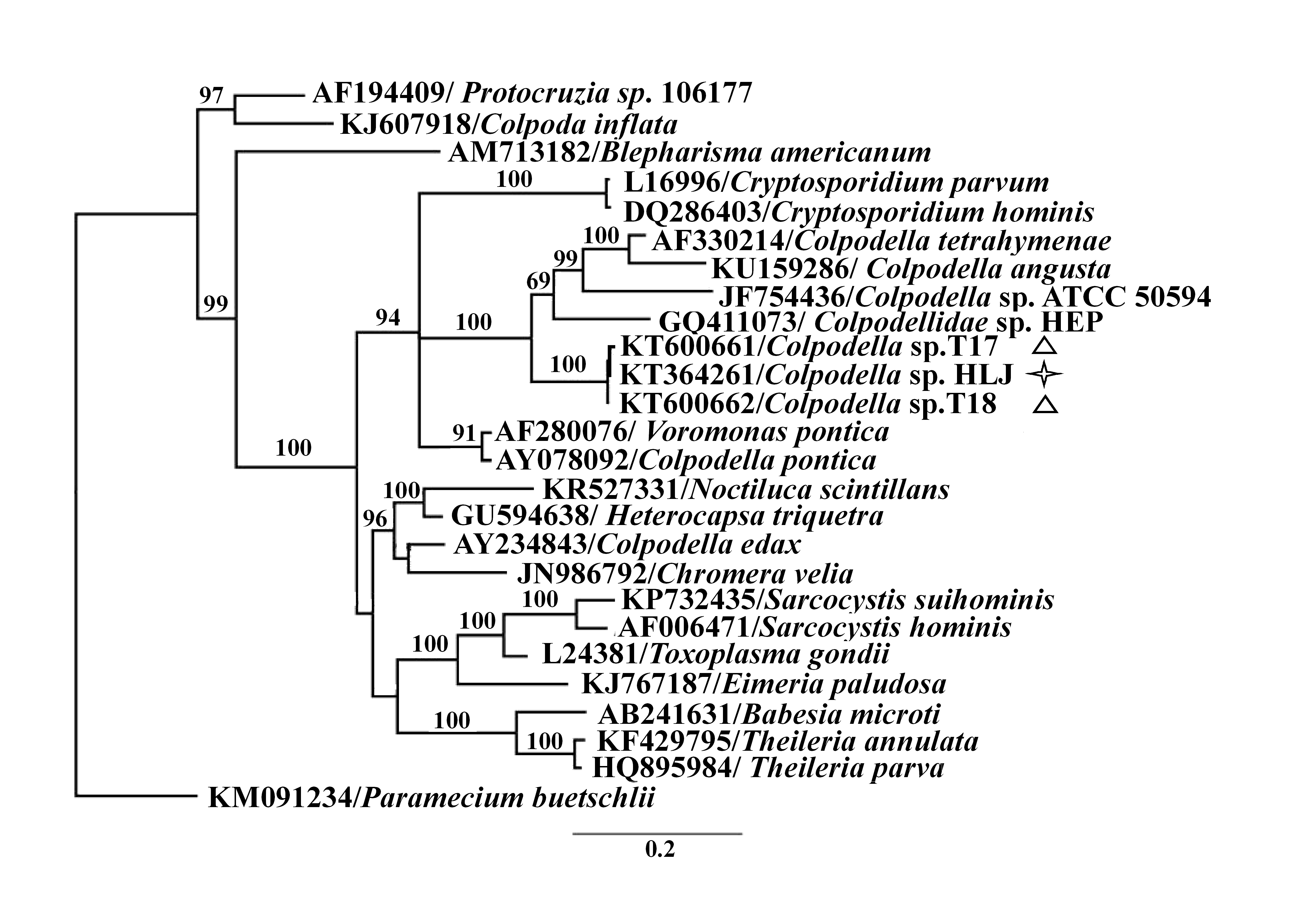

Supplement: S2 Fig — (TIF) [file pntd.0006546.s002.tif]
